# Optimization of QuEChERS Extraction for Determination of Carotenoids, Polyphenols, and Sterols in Orange Juice Using Design of Experiments and Response Surface Methodology

**DOI:** 10.3390/foods12163064

**Published:** 2023-08-15

**Authors:** Yusuke Iwasaki, Saki Yamada, Shinya Sakuma, Shunpei Kanba, Chinatsu Youda, Mizuki Ono, Rie Ito, Junzo Kamei, Hiroshi Akiyama

**Affiliations:** 1 Department of Analytical Chemistry, Hoshi University School of Pharmacy and Pharmaceutical Sciences, Tokyo 142-8501, Japan; 2 Laboratory of Biopharmaceutics and Analytical Science, Hoshi University School of Pharmacy and Pharmaceutical Sciences, Tokyo 142-8501, Japan; 3 Department of Biomolecular Pharmacology, Hoshi University School of Pharmacy and Pharmaceutical Sciences, Tokyo 142-8501, Japan; 4 Juntendo Advanced Research Institute for Health Science, Juntendo University, Tokyo 113-8421, Japan

**Keywords:** design of experiments, LC/MS/MS, QuEChERS, response surface methodology

## Abstract

Several compounds with different physical properties are present in foods, biological components, and environmental samples, and there are cases in which these must be analyzed simultaneously. However, it is difficult to extract compounds with different physical properties from the same sample using a single method. In the present study, we examined the optimal conditions for the QuEChERS extraction of several kinds of compounds from orange juice using design of experiments (DoE) and response surface methodology (RSM) to determine the optimal ratio of organic solvent to sodium chloride. We determined the optimal extraction conditions, which were within the design space, using 100% tetrahydrofuran (THF) as the extraction organic solvent and NaCl:MgSO_4_ = 75:25 as the salt. The developed LC/MS/MS method using QuEChERS extraction achieved specific detection and precise quantification. Finally, we measured the polyphenols, sterols, and carotenoids in citrus juice using the optimized QuEChERS extraction method before LC/MS/MS analysis. Most of the analytes were quantifiable in orange juice. The optimized method achieved ease of operation, the extraction of analytes from food samples in a short time (within 30 min), minimization of analytical residues, and reliability. The DoE and RSM approach may contribute to better optimization of the extraction conditions for the lowest number of experiments.

## 1. Introduction

The citrus family comprises several prominent members, including sweet orange (*Citrus sinensis*), mandarin, tangerine orange or satsuma mandarin (*Citrus reticulata* or *Citrus unshiu*), grapefruit (*Citrus paradisi*), lemon (*Citrus limon*), and lime (*Citrus aurantifolia*). Orange is a major commercial crop that contributes significantly to the fresh and processed food industries. Recently, there has been a growing interest in satsuma mandarin owing to its potential health benefits, including reduced risk of chronic diseases such as obesity, diabetes, cancer, and cardiovascular diseases [1,2,3]. Polyphenols, sterols, and carotenoids are responsible for the effectiveness against these diseases. Although it was said that polyphenols have pro-oxidant activity reacted with copper ion [4,5,6], polyphenols are used as supplements in several functional foods worldwide owing to their antioxidant activity, which can protect human organs from oxidative stress [7,8,9].

Several methods have been published for the extraction of functional molecules, such as carotenoids and polyphenols, which contain flavanones, flavones, flavanols, and hydroxycinnamic acid, from citrus samples [10,11]. The purification and extraction of many types of organic solvents from food samples require several steps, depending on the group. For example, carotenoids and sterols, which are low-polarity compounds, use n-hexane-based low-polarity organic solvents for liquid–liquid extraction from the sample [12,13]. Polyphenols such as *p*-Coumaric acid, ferulic acid, hesperidin, naringin, narirutin, and rutin, which are high polarity compounds, are extracted from citrus peel waste or fruit–vegetable juices using solid-phase extraction (SPE), including dispersive SPE or solid–liquid extraction based on an ethanolic aqueous solution [14,15,16]. In other words, it is difficult to extract compounds with different physical properties from the same sample using a single method.

A quick, easy, cheap, effective, rugged, and safe sample preparation method called QuEChERS, sometimes combined with dispersive SPE, has frequently been used for the extraction of pesticides from food samples and physiologically active substances from biological samples [17,18,19]. Phase separation and partitioning of target analytes into acetonitrile (organic phase) is typically accomplished by adding anhydrous MgSO_4_ and NaCl. While this procedure is both swift and cost effective, serving to finalize the extraction and cleanup processes, there exists a lack of comprehensive QuEChERS condition data. Specifically, the information regarding the specific organic solvents and concentration ratios of MgSO4 and NaCl needed to efficiently extract compounds of varying polarities from food samples is absent.

In the present study, we examined the optimal QuEChERS conditions for the organic solvent to sodium chloride ratio to maximize the extraction of several kinds of compounds from orange juice using design of experiments (DoE) and response surface methodology (RSM). To the best of our knowledge, no DoE- or RSM-based methods have been reported for the simultaneous extraction with QuEChERS and determination of functional compounds in food products. Finally, the developed method was applied to orange juices available in the market to evaluate analyte concentrations.

## 2. Materials and Methods

### 2.1. Chemical and Reagents

Anhydrous sodium chloride (NaCl) and magnesium sulfate (MgSO_4_) for formulations as QuEChERS components were purchased from Fujifilm Wako Pure Chemicals (Tokyo, Japan). β-Carotene, β-cryptoxanthin, campesterol, ferulic acid, stigmasterol, sitosterol, and rutin were purchased from Fujifilm Wako Pure Chemicals. *p*-Coumaric acid, diosmin, hesperidin, naringin, and narirutin were purchased from Tokyo Chemical Institute (Tokyo, Japan). Pyrogallol and 2,6-di-*tert*-butyl-4-methylphenol (BHT) used to prevent autooxidation were purchased from Fujifilm Wako Pure Chemicals (Tokyo, Japan).

All organic solvents (acetonitrile, formic acid, tetrahydrofuran, and methanol) and ultrapure HPLC- or LC/MS-grade water were purchased from Fujifilm Wako Pure Chemicals.

Orange juice was purchased from a local market in Tokyo, Japan.

### 2.2. Design of Experiments (DoE)

To study the influence of process parameters, a two-level, nine-factor (analytes), full (three-levels) factorial design was generated using the software Design-Expert^®^ 13 (Stat-Ease Inc., Minneapolis, MN, USA). The levels of the different factors were selected based on preliminary trials. The evaluated factors or independent variables were the THF to ACN (acetonitrile) ratio (X1 = THF; in the range of 0–100%) and NaCl to MgSO_4_ ratio (X2 = NaCl; in the range of 0–100%). The measured responses, or dependent variables, were the recovery ratios of each analyte (Y1 to Y12; %). Thirteen experimental runs were performed using the software, including the five central points used to measure the reproducibility of the process.

### 2.3. LC/PDA and LC/MS/MS Instrumentation and Conditions

LC/PDA analyses were performed on a 20 series containing Prominence UHPLC and an SIL-20AC autosampler (Shimadzu, Kyoto, Japan). The chromatographic separation was achieved using a SunShell C18 column (2.1 mm × 100 mm, 2.6 μm particles; ChromaNik Technologies, Osaka, Japan) fitted with a SecurityGuard ULTRA C18 (2.1 mm × 2 mm, 2 μm particles; Phenomenex, CA, USA) guard column. The mobile phase consisted of 0.1% formic acid in water and 0.1% formic acid in acetonitrile. The total run time was 45 min using the following multistep gradient: 0–15 min, 10–20% B (linear gradient); 15–16 min, 20–95% B (linear gradient); 16–20 min, 95–100% B (linear gradient); 20–35 min, 100% B (isocratic); 35–35.1 min, 100–10% B (linear gradient); 35.1–45 min, 10% B (linear gradient). The mobile phase flow rate was 0.5 mL/min, column temperature was 40 °C, and injection volume was 1 μL.

LC/MS/MS analyses were carried out utilizing an LCMS-8045 mass spectrometer equipped with a Nexera UHPLC and an SIL-30AC autosampler (Shimadzu, Kyoto, Japan). The LC conditions, including chromatographic separation, were the same as those described above for the LC/PDA method.

Electrospray ionization (ESI) was employed in both positive and negative ion modes, utilizing selective reaction monitoring (SRM) for analyte quantification. The interface voltage was set at 4.0 kV. Drying and nebulizing gases were maintained at flow rates of 10.0 and 3.0 L/min, respectively. The desolvation line temperature was set to 250 °C, while the heat block temperature was set at 400 °C.

The limit of detection (LOD) and limit of quantification (LOQ) were defined as the concentrations at which the signal-to-noise ratios (S/Ns) of 3 and >10 were observed, respectively. The evaluation of accuracy and precision was performed by repeatedly analyzing three different concentrations and determining the recovery rate.

### 2.4. Preparation of Extracts from Citrus Juice

Liquid food samples (50 μL), such as orange juice, water (400 μL), an organic solvent (THF, consisting of 3% pyrogallol and 0.1% BHT) (500 μL), and 1 mol/L HCl (50 μL), were mixed before QuEChERS extraction.

To produce the tableted QuEChERS reagent, all raw materials were pulverized and passed through a 50-mesh screen with a 300 μm aperture prior to mixing. The QuEChERS reagent, consisting of NaCl and MgSO_4_ (ratio of 75:25), was poured into the die cavity of a single-punch tableting machine (HANDTAB-100, Ichihashi Seiki, Kyoto, Japan) and compressed into tablets of 7 mm in diameter at a force of 5 kN. The tableting conditions were based on a previous study [18]. The QuEChERS tablets were added directly to each sample tube. After vigorously shaking the mixture using a vortex (10 min), it was centrifuged at 10,000× *g* for 5 min. The supernatant of the organic phase was transferred to an autosampler vial, and the sample was injected for LC/PDA or LC/MS/MS analysis.

### 2.5. Statistical Analysis

All results are presented as the mean ± S.D. of three or six independent experiments. Data were performed using Ekuseru-Toukei 2015 (Social Survey Research Information Co., Ltd., Tokyo, Japan).

## 3. Results and Discussion

### 3.1. Examination of LC/PDA and LC/MS/MS Conditions to Measure the Polyphenols, Phytosterols, and Carotenoids

**Figure 1:**
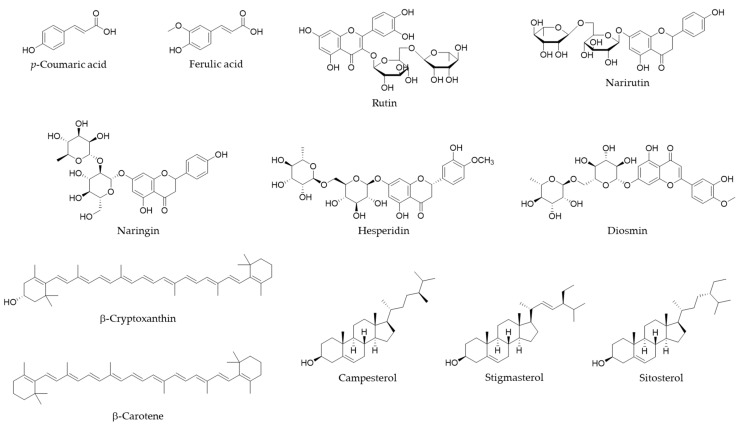
Chemical structures of the analytes.

**Figure 2:**
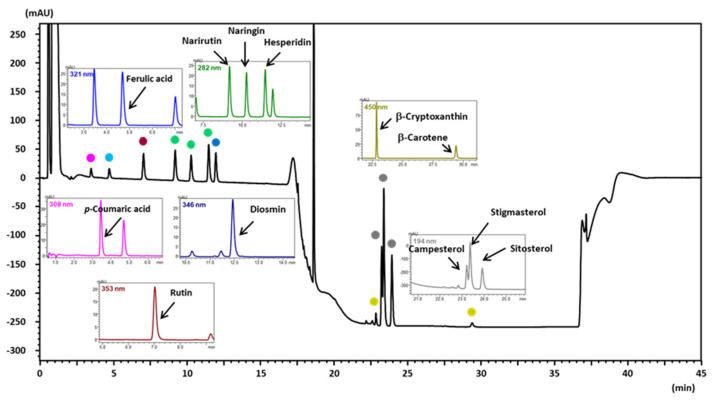
Typical standard chromatograms for each analyte solution obtained using LC/PDA. Black line represents the total wavelength chromatogram.

In the first experiment, PDA and MS/MS conditions were optimized to examine the separation conditions for the simultaneous analysis of highly polar and less polar compounds (Figure 1) in citrus juice using an LC instrument. Figure S1 shows the maximum absorption wavelength, which represents the chemical structures of the analytes. When analyzing polyphenols using reversed phase chromatography, it is necessary to analyze with a high proportion of the aqueous phase of the mobile phase, whereas, for carotenoids, it is necessary to excessively increase the proportion of the organic phase. Therefore, when only analyzing carotenoids, there is a method of using normal phase chromatography [20]; however, under these conditions, it becomes difficult to analyze polyphenols simultaneously. We considered a method of simultaneously analyzing polyphenols, sterols, and carotenoids by using a short ODS column and increasing the flow rate. As a result, because all analytes could be separated and detected using LC/PDA (Figure 2), we designed the QuEChERS extraction method described in the next experimental section.

On the other hand, the SRM conditions necessary to quantify all analytes from citrus juice were examined with mass spectrometry using flow injection analysis. All polyphenols were detected in the negative-ion ESI mode. Sterols detected in the positive-ion mode selected dehydrated ions as precursor ions. The details of the UV wavelength and SRM parameters for each analyte are listed in Table S1. The optimized LC/PDA and MS/MS conditions allowed us to achieve reliable and simultaneous analysis of polyphenols, sterols, and carotenoids in citrus juice. By employing a short ODS column and increasing the flow rate, we could efficiently separate and detect the analytes, overcoming the challenges posed by their different polarities and chromatographic requirements.

### 3.2. Optimization of QuEChERS Extraction with Response Surface Methodology

DoE is an essential tool to better understand optimal tablet formulations. RSM is a method for modeling and optimizing the conditions that require the lowest number of experiments to obtain appropriate results [21,22]. DoE and RSM are commonly used to obtain optimized formulations considering a wide range of factors that can affect the target product profile, as experiments are set up in an efficient and precise way [23,24].

We examined several types of organic solvents that facilitate mixing with aqueous phases, such as methanol, ethanol, acetone, ACN, and THF, to completely separate the two phases after adding the QuEChERS reagent. A preliminary examination showed that ACN and THF could be clearly separated into aqueous and organic phases using the QuEChERS reagent. Next, we selected the organic solvents (ACN and THF) and salt ratios as the extraction factors for the independent variables. Various extraction parameters, such as the THF to ACN concentration ratio (X1 = THF; in the range of 0–100%) and NaCl to MgSO_4_ concentration ratio (X2 = NaCl; in the range of 0–100%), were optimized using a full (3 level) factorial design. A response surface fractional factorial design in randomized order with five center points was designed with two independent and 13 dependent variables. Table S2 summarizes the printing parameters and results obtained for the analytes recoveries in each run.

The model suggested by the program is presented in Table S3. The first step in mathematical modeling involved fitting the experimental data to an appropriate model. Based on the regression coefficients, the quadratic model showed the smallest p-value and the biggest r-squares compared to other models, such as the liner, two-factor interaction (2FI), and the cubic model. However, the cubic model was found to be aliased. Among the models considered, the quadratic model demonstrated the maximum adjusted R^2^ and predicted R^2^, making it the most suitable choice. Additionally, to be considered suitable, a model must exhibit a nonsignificant lack of fit. The lack of fit p-values for the linear and 2FI models were below 0.05, except for carotene, indicating a significant lack of fit test for the quadratic model was not significant (*p*-value > 0.05), suggesting that it adequately represented the experimental data in this research. Therefore, the quadratic model was selected for further analysis.

The significance of the quadratic model terms was determined using the analysis of variance (ANOVA), as shown in Table S4. The R^2^ values for all analytes were above 0.80, indicating that the model obtained was highly significant and adequate. Generally, an acceptable model should have an R^2^ value of at least 0.75 [25,26]. However, it is essential to note that adding additional variables to the model will always increase the R^2^ value, regardless of whether these variables are statistically significant or not. Therefore, it was said that a high R^2^ value does not necessarily imply the adequacy of the model [27,28].

**Figure 3:**
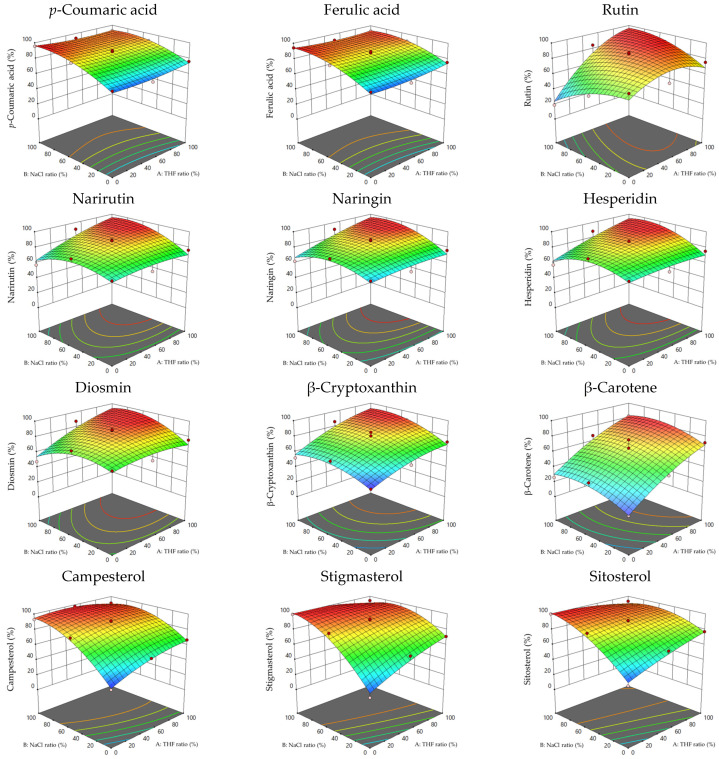
Response surface plots (3D) of THF ratio (X1) and NaCl ratio (X2) on extraction recovery.

Figure 3 shows various contour plots depending on the relationship between the variables. Each analyte was divided into four groups using simulated RSM. All analytes were effectively extracted using a higher THF ratio as the organic solvent when QuEChERS extraction was used. Although sterols tended to increase the extraction recovery with high NaCl concentrations, only rutin decreased the extraction recovery. In other words, the RSM results showed that good extraction of only sterols was possible by setting the NaCl concentration to 100% and THF concentration to 0%.

**Figure 4:**
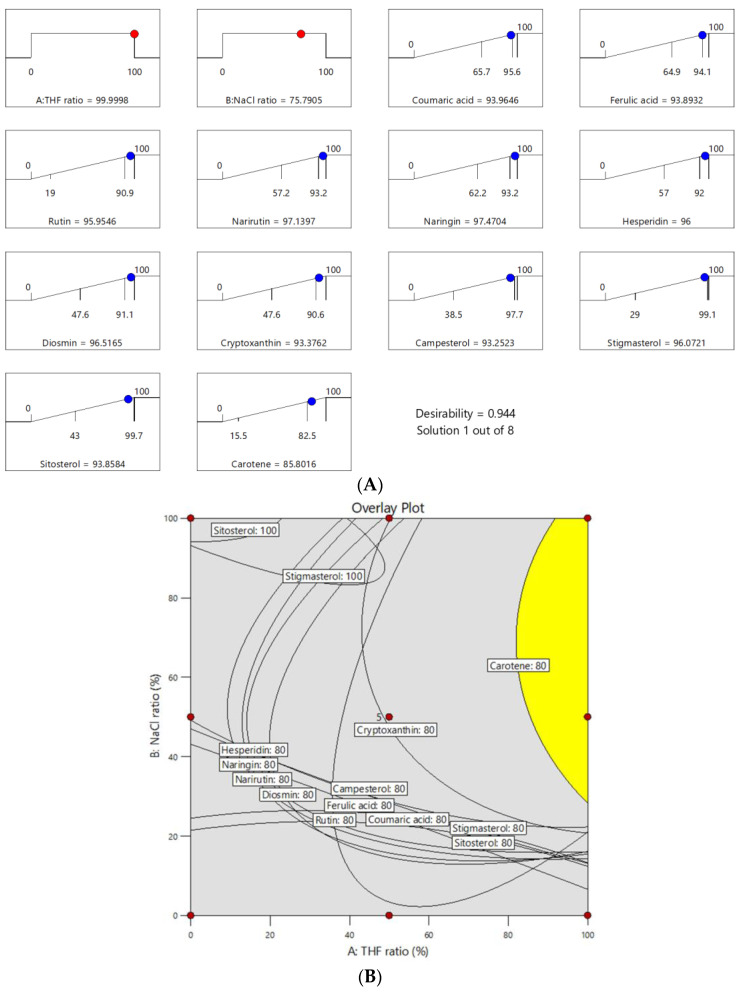
Numerical optimization ramps predicting the optimum conditions (**A**) and overlay plot (**B**) for maximum recovery rate.

We considered the need for optimal conditions for easy sample preparation, a high extraction ratio, and a high-precision method to extract analytes from food samples. The results were reinstated using numerical optimization ramps to predict the optimum conditions and an overlay plot of the maximum recovery rate (Figure 4). The yellow space represents the acceptance area with a recovery rate of over 80%. We determined the optimal extraction conditions, which were contained in the design space, using 100% THF as the organic extraction solvent and NaCl:MgSO_4_ = 75:25 as the salt.

### 3.3. Assessment of Recovery Rate with LC/PDA and LC/MS/MS Measurement Using Optimized QuEChERS Extraction Method

We determined that the QuEChERS extraction conditions were 100% THF as the extraction solvent and NaCl:MgSO_4_ = 75:25 as the salt to saturate the ionic strength in the aqueous phase. The LC/PDA and LC/MS/MS methods were validated. The LC/PDA recovery results indicated that the developed method, according to the optimal QuEChERS extraction, yielded a good absolute recovery rate (Table 1A). However, campesterol had a low recovery rate with LC/MS/MS (Table 1B,C). Ion suppression by other components may be the main reason for the low recovery rate of campesterol, although samples extracted using the QuECHERS method were highly reproducible, even without the use of internal standards.

The normalized areas of the chromatographic peaks were linear in the analyzed concentration range, with regression coefficients exceeding 0.99, allowing for further quantification studies. Our LC/MS/MS method using QuEChERS extraction achieved specific detection and precise quantification (Table 2).

Several techniques, such as SPE, liquid-phase microextraction (LPME), solid-phase microextraction (SPME), and dispersive liquid–liquid microextraction (DLLME), have been frequently used in the pretreatment process for analytes extraction and concentration [29]. Green extraction techniques aim to provide greener and more sustainable alternatives to classical methods by improving the selectivity and sensitivity of analytical methods while simultaneously reducing the harmful side effects for both the operator and the environment [30]. The QuEChERS extraction method we optimized made it possible to extract compounds with various physical properties in a short time with good reproducibility, and the cost of preprocessing was also reduced compared to methods such as solid-phase extraction. Furthermore, our method uses only a small amount of solvent in preprocessing and is therefore considered to contribute to green extraction techniques.

### 3.4. Determination of Polyphenols, Sterols, and Carotenoids in Citrus Juice with QuEChERS Extraction and LC/MS/MS Analysis

We measured the polyphenols, sterols, and carotenoids in citrus juice sold at a supermarket in Japan using the optimized QuEChERS extraction method prior to LC/MS/MS analysis (Table 3). Most of the analytes, except for ferulic acid and campesterol, whose LOQs were very low and had a low recovery rate, were quantified in orange juice. Almost the same quantitative values were obtained when the analytes were compared among the 100% fruit juice ratios. In particular, high concentrations of hesperidin and narirutin were detected. In a previous study, rutin, narirutin, and hesperidin were detected in orange juice. Although the authors used SPE to purify and extract polyphenols from orange juice, our optimal QuEChERS method yielded the same or higher quantification value [14,31,32]. Sample No. 8, which is sold as a functional food in Japan to prevent osteoporosis, was detected with the high concentration of cryptoxanthin. Because the QuEChERS material was made of NaCl and MgSO_4_, our optimal extraction process was a cheap and easy method for liquid sample extraction. Moreover, the method we optimized allows for the extraction of analytes from food samples in a short time (within 30 min), even when compared to past publications. However, this study had some limitations. Highly accurate values were obtained without using an internal standard. Therefore, for more reliable analysis, it is better to prepare an internal standard for each substance to be analyzed.

## 4. Conclusions

Many compounds with different physical properties are present in foods, biological components, and environmental samples, and there are cases in which they must be analyzed simultaneously. Various sample pretreatment methods exist; however, it is difficult to develop a sample pretreatment method that can handle various physical properties. The traditional methods were used liquid–liquid extraction and/or SPE to extract each analyte. Our optimal QuEChERS conditions, using 100% THF as the extraction organic solvent and NaCl:MgSO_4_ = 75:25 as the salt, achieved good precision and provided an easy preparation method to extract analytes from orange juice. The optimized method achieved easy operation, the extraction of analytes from food samples in a short time, minimization of analytical residues, and reliability. In contrast to previous methods that utilized various organic solvents and multiple extraction and purification steps from food samples, the optimized QuEChERS method employed only 500 μL of organic solvent in a single extraction step to efficiently extract analytes from orange juice.

The implementation of DoE and RSM played a crucial role in optimizing the extraction conditions while minimizing the number of experiments required. This approach allowed for systematic and efficient exploration of the experimental parameter space, resulting in the identification of optimal conditions with a reduced number of trials. Overall, the successful application of the optimized method and the utilization of DoE and RSM not only enhanced the efficiency and reliability of the analytical process but also contributed to the advancement of sustainable and environmentally friendly practices by significantly reducing the consumption of organic solvents and overall waste.

## Figures and Tables

**Table 1:** Intra- and inter-day recovery of polyphenols, sterols, and carotenoids with LC/PDA and LC/MS/MS after QuEChERS extraction. (**A**) Intra-day assay for LC/PDA (*n* = 6), (**B**) Intra-day assay for LC/MS/MS (*n* = 6), (**C**) Inter-day assay for LC/MS/MS (*n* = 3).

(A)									
Analytes	Concentration (μM)	Accuracy (%)	Precision (%)	Concentration (μM)	Accuracy (%)	Precision (%)			
*p*-Coumaric acid	10	94.3	3.92	25	110	8.36			
Ferulic acid	10	87.8	8.36	25	93.8	6.17			
Rutin	10	88.5	9.71	25	94.0	11.4			
Narirutin	200	90.1	2.32	500	95.5	4.03			
Naringin	10	97.5	10.9	25	96.6	2.44			
Hesperidin	1000	86.8	4.29	2500	91.9	3.93			
Diosmin	10	92.5	3.81	25	92.9	6.01			
β-Cryptoxanthin	10	107	4.14	25	99.1	2.80			
Campesterol	100	111	7.97	250	105	4.10			
Stigmasterol	100	107	3.41	250	103	2.37			
Sitosterol	1000	82.7	4.95	2000	87.3	8.17			
β-Carotene	100	97.2	11.5	250	106	6.78			
**(B)**									
**Analytes**	**Concentration (μM)**	**Accuracy (%)**	**Precision (%)**	**Concentration (μM)**	**Accuracy (%)**	**Precision (%)**	**Concentration (μM)**	**Accuracy (%)**	**Precision (%)**
*p*-Coumaric acid	5	92.6	16.0	10	90.3	9.71	20	103	11.4
Ferulic acid	50	111	17.5	100	110	4.57	200	105	9.82
Rutin	5	86.9	18.9	10	98.3	9.74	20	95.7	8.83
Narirutin	100	99.8	17.9	200	91.8	12.2	400	98.8	11.5
Naringin	0.5	108	15.3	1	96.7	7.85	2	103	8.92
Hesperidin	500	92.2	16.3	1000	87.5	16.4	2000	94.1	11.9
Diosmin	0.5	97.4	3.76	1	92.2	9.67	2	96.8	3.85
β-Cryptoxanthin	0.5	84.2	11.6	1	86.1	10.5	2	98.1	12.6
Campesterol	25	65.0	11.2	50	85.9	3.73	100	73.6	4.02
Stigmasterol	25	73.6	11.6	50	87.9	10.5	100	102	8.48
Sitosterol	100	54.3	10.9	200	80.7	8.08	400	86.0	7.15
β-Carotene	5	96.8	5.68	10	104	14.8	20	96.0	2.83
**(C)**									
**Analytes**	**Concentration (μM)**	**Accuracy (%)**	**Precision (%)**	**Concentration (μM)**	**Accuracy (%)**	**Precision (%)**	**Concentration (μM)**	**Accuracy (%)**	**Precision (%)**
*p*-Coumaric acid	5	96.4	3.43	10	102	6.97	20	107	8.49
Ferulic acid	50	109	12.7	100	102	8.65	200	104	3.47
Rutin	5	93.0	9.19	10	95.1	5.23	20	97.8	3.53
Narirutin	100	99.5	10.4	200	95.8	6.72	400	99.6	4.94
Naringin	0.5	98.4	8.19	1	94.8	5.50	2	104	3.13
Hesperidin	500	98.0	8.88	1000	90.7	13.3	2000	98.7	4.36
Diosmin	0.5	98.5	4.67	1	104	6.14	2	98.0	1.31
β-Cryptoxanthin	0.5	89.8	6.20	1	92.2	1.99	2	95.9	1.60
Campesterol	25	75.6	9.28	50	81.4	3.32	100	81.9	6.81
Stigmasterol	25	85.2	8.44	50	91.8	1.08	100	99.5	4.55
Sitosterol	100	69.7	13.3	200	84.4	12.0	400	91.0	9.37
β-Carotene	5	96.5	5.74	10	96.5	7.32	20	99.1	4.72

**Table 2:** Regression equations, linear ranges, LOD, and LOQ of 12 analytes determined with LC/MS/MS. LOD and LOQ were defined as the concentrations for which S/N = 3 and >10 were observed, respectively.

Analytes	LOD (μM)	LOQ (μM)	Calibration Range	Linearity
*p*-Coumaric acid	1	2.5	2.5–50	0.999
Ferulic acid	10	25	25–500	0.999
Rutin	0.5	2.5	2.5–50	0.999
Narirutin	1	50	50–1000	0.999
Naringin	0.1	0.5	0.5–10	0.999
Hesperidin	1	250	250–2500	0.999
Diosmin	0.1	0.25	0.25–5	0.999
β-Cryptoxanthin	0.1	0.25	0.25–10	0.999
Campesterol	10	50	50–250	0.999
Stigmasterol	10	50	50–500	0.999
Sitosterol	10	100	100–500	0.999
β-Carotene	0.5	5	5–100	0.999

LOD and LOQ were defined as the concentrations for which S/N = 3 and >10 were observed, respectively.

**Table 3:** Quantitative determination of analytes in commercially available orange juice.

Sample No.	1	2	3	4	5	6	7	8
Manufacturer	USA	USA	USA	USA	USA	Japan	Japan	Japan
Fruit juice ratio	30	100	100	100	100	100	100	100
Analytes	1	2	3	4	5	6	7	8
*p*-Coumaric acid	N.D.	Trace (0.84 ± 0.07)	Trace (1.17 ± 0.26)	Trace (1.18 ± 0.32)	Trace (2.37 ± 0.27)	N.D.	Trace (1.16 ± 0.30)	Trace (1.64 ± 0.10)
Ferulic acid	N.D.	N.D.	N.D.	N.D.	N.D.	N.D.	N.D.	N.D.
Rutin	Trace (0.65 ± 0.04)	4.95 ± 0.07	5.62 ± 0.26	5.12 ± 0.09	5.80 ± 0.21	5.83 ± 0.18	5.57 ± 0.08	20.04 ± 0.79
Narirutin	Trace (17.7 ± 0.5)	100.4 ± 2.2	120.2 ± 5.8	101.0 ± 2.8	110.7 ± 3.8	109.4 ± 5.1	103.8 ± 2.9	347.0 ± 17.6
Naringin	N.D.	N.D.	N.D.	N.D.	N.D.	N.D.	0.44 ± 0.06	N.D.
Hesperidin	Trace (93 ± 3)	916 ± 8	1054 ± 29	883 ± 20	862 ± 25	837 ± 27	680 ± 13	1096 ± 34
Diosmin	Trace (0.11 ± 0.01)	0.65 ± 0.01	0.72 ± 0.01	0.64 ± 0.02	0.62 ± 0.03	0.61 ± 0.01	0.42 ± 0.01	0.38 ± 0.01
β-Cryptoxanthin	Trace (0.10 ± 0.01)	0.68 ± 0.04	0.70 ± 0.03	0.55 ± 0.03	0.68 ± 0.04	1.10 ± 0.05	0.92 ± 0.04	8.62 ± 0.06
Stigmasterol	N.D.	N.D.	N.D.	N.D.	N.D.	N.D.	N.D.	N.D.
Sitosterol	N.D.	112.6 ± 7.3	124.0 ± 9.8	110.5 ± 10.0	97.7 ± 1.8	111.7 ± 0.8	109.3 ± 7.4	148.2 ± 5.4
β-Carotene	Trace (0.62 ± 0.03)	6.04 ± 0.12	6.50 ± 0.07	5.55 ± 0.03	8.08 ± 0.23	8.23 ± 0.13	7.27 ± 0.29	57.11 ± 0.79

Concentration units are μM. N.D.: not detected (below the LOD). Trace levels were above the LOD and below the LOQ.

## Data Availability

Data are contained within the article and Supplementary Material.

## Supplementary Materials

The following supporting information can be downloaded at: https://www.mdpi.com/article/10.3390/foods12163064/s1, Figure S1: Chemical structures and UV spectra of the analytes; Table S1: UV wavelengths and SRM parameters for each analyte; Table S2: Composition of batches and experimental results. Mean ± S.D. (*n* = 3); Table S3: Fitting models of each analyte; Table S4: ANOVA and fit statistics data for each analyte.
