# Supplementary material for: Optimization of QuEChERS Extraction for Determination of Carotenoids, Polyphenols, and Sterols in Orange Juice Using Design of Experiments and Response Surface Methodology
Source: Foods. 2023 Aug 15;12(16):3064. doi: 10.3390/foods12163064 (PMC10453318; doi:10.3390/foods12163064)
Supplement: Supplementary file 1 [file foods-12-03064-s001.zip › foods-2530315-supplementary.pdf]

Figure S1. Chemical structures and UV spectra of the analytes.

p-Coumaric acid	Ferulic acid	Rutin	Narirutin
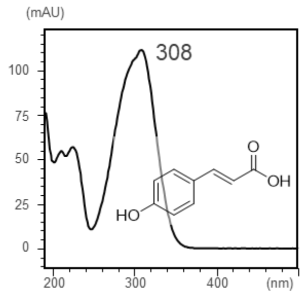	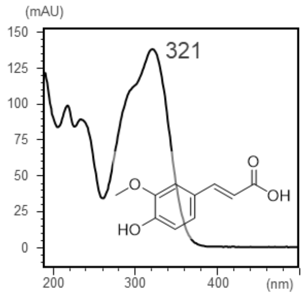	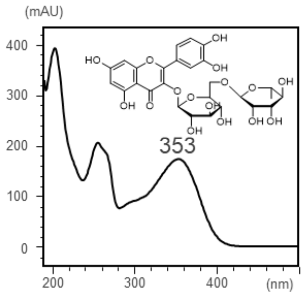	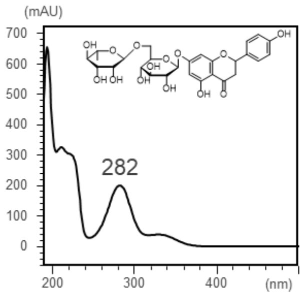
**Naringin**	**Hesperidin**	**Diosmin**	**β-Cryptoxanthin**
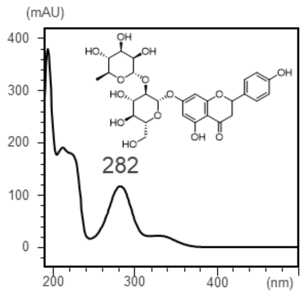	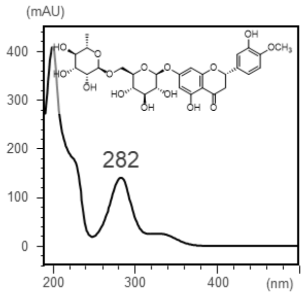	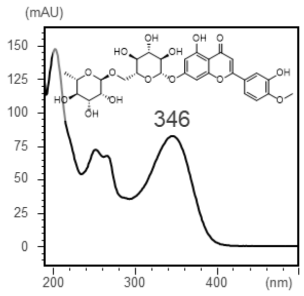	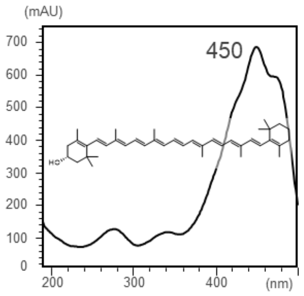
**β-Carotene**	**Campesterol**	**Stigmasterol**	**Sitosterol**
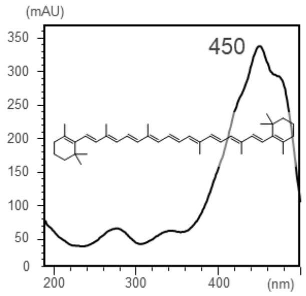	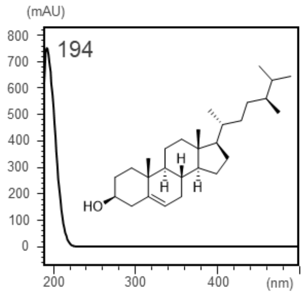	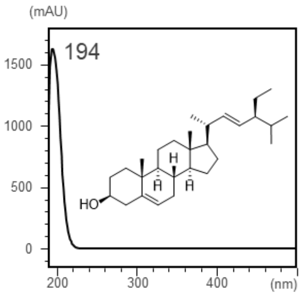	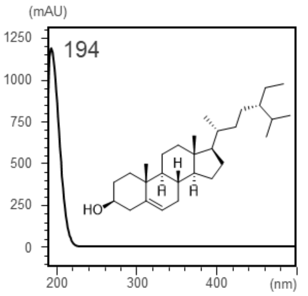

Table S1 UV wavelengths and SRM parameters for each analyte

Analytes	Molecular weight	Wavelength	Polarity	Precursor ion	Product ion	Q1 pre bias	Collision energy	Q3 pre bias
p-Coumaric acid	164.16	308	Negative	162.8	119.0	18	16	21
Ferulic acid	194.19	321	Negative	193.1	134.0	20	18	24
Rutin	610.52	353	Negative	609.1	300.0	30	39	30
Narirutin	580.54	282	Negative	579.1	271.0	22	25	17
Naringin	580.54	282	Negative	579.1	270.9	30	35	27
Hesperidin	610.57	282	Negative	608.9	301.0	30	25	19
Diosmin	608.55	346	Negative	607.0	299.0	30	27	19
β-Cryptoxanthin	552.89	450	Positive	552.5	460.2	-24	-16	-16
Campesterol	400.69	194	Positive	383.5	147.0	-15	-25	-29
Stigmasterol	412.70	194	Positive	395.2	82.9	-15	-22	-17
Sitosterol	414.72	194	Positive	397.2	147.0	-12	-25	-28
β-Carotene	536.89	450	Positive	536.2	444.2	-22	-15	-16

Table S2 Composition of batches and experimental results. Mean ± S.D. (n = 3).

	Independent		Dependent											
Run No.	THF ratio (%)	NaCl ratio (%)	Coumaric acid	Ferulic acid	Rutin	Narirutin	Naringin	Hesperidin	Diosmin	Cryptoxanthin	Campesterol	Stigmasterol	Sitosterol	Carotene
1	50	50	87.7 ± 2.0	86.6 ± 3.3	85.0 ± 1.4	86.8 ± 2.4	86.9 ± 1.1	86.2 ± 2.7	86.1 ± 2.0	85.2 ± 4.8	89.6 ± 4.0	89.0 ± 1.5	89.1 ± 1.9	75.6 ± 4.7
2	0	50	88.6 ± 3.6	86.1 ± 3.9	48.5 ± 2.3	80.1 ± 3.1	80.1 ± 2.1	80.3 ± 3.2	77.0 ± 3.4	63.8 ± 4.3	83.8 ± 3.0	89.2 ± 1.3	89.2 ± 2.2	36.8 ± 1.6
3	50	0	65.7 ± 2.7	64.9 ± 3.5	64.2 ± 1.8	64.9 ± 2.3	64.5 ± 2.0	64.4 ± 2.9	65.0 ± 2.8	59.1 ± 4.7	58.8 ± 6.9	61.5 ± 4.9	67.7 ± 1.6	45.8 ± 5.8
4	100	0	76.2 ± 3.8	75.5 ± 5.2	75.4 ± 3.9	76.5 ± 3.7	76.1 ± 3.1	75.1 ± 4.6	75.8 ± 4.2	72.8 ± 7.9	66.7 ± 10.5	70.9 ± 6.9	77.1 ± 2.7	71.8 ± 7.1
5	100	50	90.3 ± 2.1	90.2 ± 2.8	89.3 ± 2.0	89.9 ± 1.5	91.7 ± 1.4	90.5 ± 2.4	89.8 ± 1.3	88.3 ± 5.7	88.4 ± 3.9	91.3 ± 0.3	89.6 ± 0.3	82.5 ± 4.9
6	50	100	93.6 ± 1.7	91.6 ± 2.3	84.7 ± 1.3	91.0 ± 1.6	90.7 ± 1.0	88.2 ± 1.6	87.4 ± 0.9	86.2 ± 5.2	97.7 ± 5.5	97.2 ± 1.9	95.8 ± 0.8	67.3 ± 3.5
7	50	50	88.8 ± 1.8	87.7 ± 2.2	86.2 ± 1.4	88.6 ± 1.0	87.5 ± 1.4	86.7 ± 2.1	88.4 ± 2.0	80.6 ± 3.3	89.5 ± 5.4	90.8 ± 2.5	90.1 ± 1.7	64.8 ± 2.8
8	0	0	71.2 ± 1.9	70.4 ± 2.2	68.7 ± 1.7	70.2 ± 1.2	69.9 ± 1.2	69.7 ± 1.8	69.3 ± 1.9	47.6 ± 1.0	38.5 ± 2.8	29.0 ± 8.7	43.0 ± 8.9	15.5 ± 6.5
9	50	50	89.3 ± 1.4	88.5 ± 2.1	86.4 ± 0.2	89.3 ± 0.6	88.7 ± 0.5	87.8 ± 2.0	88.1 ± 1.2	80.1 ± 3.2	91.1 ± 5.8	92.1 ± 0.4	91.0 ± 0.6	63.3 ± 2.5
10	100	100	92.1 ± 2.9	92.3 ± 3.8	90.9 ± 2.9	93.2 ± 3.2	93.2 ± 3.3	91.7 ± 2.6	91.1 ± 3.0	90.6 ± 7.5	90.6 ± 4.9	93.8 ± 3.1	92.9 ± 3.0	81.7 ± 8.5
11	50	50	90.1 ± 2.3	89.4 ± 3.1	87.8 ± 2.8	88.8 ± 3.4	89.5 ± 2.9	88.0 ± 3.8	89.0 ± 2.9	78.9 ± 4.3	91.3 ± 5.5	93.3 ± 1.5	91.5 ± 1.1	60.8 ± 2.9
12	0	100	95.6 ± 3.2	94.1 ± 3.9	19.0 ± 1.0	57.2 ± 2.7	62.2 ± 1.7	57.3 ± 2.3	47.6 ± 1.8	52.1 ± 11.7	93.0 ± 3.3	99.1 ± 1.2	99.7 ± 0.3	26.0 ± 5.9
13	50	50	88.2 ± 1.9	87.9 ± 2.6	86.7 ± 1.8	87.9 ± 3.0	87.8 ± 0.9	85.8 ± 2.1	87.2 ± 2.1	75.3 ± 4.1	90.7 ± 4.7	91.0 ± 1.6	90.0 ± 0.7	54.5 ± 2.3

Table S3 Fitting models of each analyte.

(1) *p*-Coumaric acid

Source	Sequential p-value	Lack of Fit p-value	Adjusted R^2	Predicted R^2	
Linear	0.0004	0.0017	0.7486	0.6096	
2FI	0.3765	0.0015	0.7452	0.5107	
Quadratic	0.0021	0.0214	0.9437	0.7258	Suggested
Cubic	0.0832	0.0362	0.9709	-0.0012	Aliased

(2) Ferulic acid

Source	Sequential p-value	Lack of Fit p-value	Adjusted R^2	Predicted R^2	
Linear	0.0004	0.0026	0.7471	0.6233	
2FI	0.4736	0.0021	0.7354	0.5368	
Quadratic	0.0027	0.0262	0.9372	0.7188	Suggested
Cubic	0.2253	0.0195	0.9516	-0.8367	Aliased

(3) Rutin

Source	Sequential p-value	Lack of Fit p-value	Adjusted R^2	Predicted R^2	
Linear	0.0447	< 0.0001	0.3553	-0.2401	
2FI	0.0433	< 0.0001	0.5561	-0.3992	
Quadratic	0.0135	0.0001	0.8333	0.0360	Suggested
Cubic	0.0012	0.0056	0.9844	0.3339	Aliased

(4) Narirutin

Source	Sequential p-value	Lack of Fit p-value	Adjusted R^2	Predicted R^2	
Linear	0.0863	< 0.0001	0.2648	-0.3800	
2FI	0.1334	0.0001	0.3728	-1.1315	
Quadratic	0.0213	0.0004	0.7315	-0.5735	Suggested
Cubic	< 0.0001	0.7517	0.9940	0.9884	Aliased

(5) Naringin

Source	Sequential p-value	Lack of Fit p-value	Adjusted R^2	Predicted R^2	
Linear	0.0527	0.0002	0.3339	-0.2253	
2FI	0.1647	0.0002	0.4099	-0.9409	
Quadratic	0.0129	0.0011	0.7810	-0.2740	Suggested
Cubic	< 0.0001	0.9575	0.9926	0.9952	Aliased

(6) Hesperidin

Source	Sequential p-value	Lack of Fit p-value	Adjusted R^2	Predicted R^2	
Linear	0.0905	0.0001	0.2579	-0.3964	
2FI	0.1289	0.0001	0.3708	-1.1242	
Quadratic	0.0125	0.0007	0.7689	-0.3465	Suggested
Cubic	0.0001	0.3119	0.9916	0.8944	Aliased

(7) Diosmin

Source	Sequential p-value	Lack of Fit p-value	Adjusted R^2	Predicted R^2	
Linear	0.1154	< 0.0001	0.2209	-0.4844	
2FI	0.1059	0.0001	0.3629	-1.2299	
Quadratic	0.0127	0.0007	0.7649	-0.3731	Suggested
Cubic	< 0.0001	0.5497	0.9932	0.9644	Aliased

(8) β-Cryptoxanthin

Source	Sequential p-value	Lack of Fit p-value	Adjusted R^2	Predicted R^2	
Linear	0.0017	0.0318	0.6643	0.4059	
2FI	0.4427	0.0271	0.6520	-0.2693	
Quadratic	0.0087	0.1684	0.8845	0.5093	Suggested
Cubic	0.0750	0.5352	0.9426	0.6827	Aliased

(9) Campesterol

Source	Sequential p-value	Lack of Fit p-value	Adjusted R²	Predicted R²	
Linear	0.0025	< 0.0001	0.6371	0.3179	
2FI	0.1449	< 0.0001	0.6857	-0.1741	
Quadratic	< 0.0001	0.0187	0.9864	0.9282	Suggested
Cubic	0.0127	0.1667	0.9967	0.9320	Aliased

(10) Stigmasterol

Source	Sequential p-value	Lack of Fit p-value	Adjusted R²	Predicted R²	
Linear	0.0045	0.0003	0.5934	0.1872	
2FI	0.0506	0.0005	0.7114	-0.0761	
Quadratic	0.0015	0.0080	0.9416	0.6866	Suggested
Cubic	0.0146	0.0554	0.9849	0.5280	Aliased

(11) Sitosterol

Source	Sequential p-value	Lack of Fit p-value	Adjusted R²	Predicted R²	
Linear	0.0039	< 0.0001	0.6049	0.1879	
2FI	0.0200	0.0002	0.7671	0.1607	
Quadratic	0.0029	0.0029	0.9436	0.6825	Suggested
Cubic	0.0026	0.0792	0.9927	0.7925	Aliased

(12) β-Carotene

Source	Sequential p-value	Lack of Fit p-value	Adjusted R²	Predicted R²	
Linear	< 0.0001	0.3210	0.8110	0.7028	
2FI	0.9758	0.2589	0.7900	0.3442	
Quadratic	0.0369	0.7066	0.8948	0.7721	Suggested
Cubic	0.5016	0.7132	0.8883	0.7319	Aliased

Table S4 ANOVA and fit statistics data for each analyte.

(1) *p*-Coumaric acid

Source	Sum of Squares	df	Mean Square	F-value	p-value	
**Quadratic model**	950.54	5	190.11	41.26	< 0.0001	significant
A-THF ratio	1.71	1	1.71	0.3704	0.5620	
B-NaCl ratio	775.21	1	775.21	168.24	< 0.0001	
AB	18.06	1	18.06	3.92	0.0882	
A^2	14.83	1	14.83	3.22	0.1159	
B^2	154.64	1	154.64	33.56	0.0007	
**Residual**	32.25	7	4.61			
Lack of Fit	28.75	3	9.58	10.93	0.0214	significant
Pure Error	3.51	4	0.8770			
**Cor Total**	982.79	12				
**Std. Dev.**	2.15		**R^2**	0.9672		
**Mean**	85.95		**Adjusted R^2**	0.9437		
**C.V. %**	2.50		**Predicted R^2**	0.7258		
			**Adeq. Precision**	18.5033		

(2) Ferulic acid

Source	Sum of Squares	df	Mean Square	F-value	p-value	
**Quadratic model**	929.86	5	185.97	36.84	< 0.0001	significant
A-THF ratio	9.13	1	9.13	1.81	0.2207	
B-NaCl ratio	752.64	1	752.64	149.09	< 0.0001	
AB	11.90	1	11.90	2.36	0.1685	
A^2	15.86	1	15.86	3.14	0.1196	
B^2	155.50	1	155.50	30.80	0.0009	
**Residual**	35.34	7	5.05			

Lack of Fit	31.07	3	10.36	9.71	0.0262	significant
Pure Error	4.27	4	1.07			
**Cor Total**	965.20	12				
**Std. Dev.**	2.25		**R^2**	0.9634		
**Mean**	85.02		**Adjusted R^2**	0.9372		
**C.V. %**	2.64		**Predicted R^2**	0.7188		
			**Adeq Precision**	16.9349		

(3) Rutin

Source	Sum of Squares	df	Mean Square	F-value	p-value	
**Quadratic model**	4696.07	5	939.21	13.00	0.0020	significant
A-THF ratio	2376.06	1	2376.06	32.88	0.0007	
B-NaCl ratio	31.28	1	31.28	0.4329	0.5316	
AB	1062.76	1	1062.76	14.71	0.0064	
A^2	568.60	1	568.60	7.87	0.0263	
B^2	213.80	1	213.80	2.96	0.1291	
**Residual**	505.82	7	72.26			
Lack of Fit	501.77	3	167.26	165.27	0.0001	significant
Pure Error	4.05	4	1.01			
**Cor Total**	5201.89	12				
**Std. Dev.**	8.50		**R^2**	0.9028		
**Mean**	74.83		**Adjusted R^2**	0.8333		
**C.V. %**	11.36		**Predicted R^2**	0.0360		
			**Adeq Precision**	12.5368		

(4) Narirutin

Source	Sum of Squares	df	Mean Square	F-value	p-value	
**Quadratic model**	1307.33	5	261.47	7.54	0.0097	significant

A-THF ratio	452.40	1	452.40	13.04	0.0086	
B-NaCl ratio	148.01	1	148.01	4.27	0.0777	
AB	220.52	1	220.52	6.36	0.0397	
A^2	33.27	1	33.27	0.9591	0.3600	
B^2	305.70	1	305.70	8.81	0.0208	
**Residual**	242.82	7	34.69			
Lack of Fit	239.07	3	79.69	85.05	0.0004	significant
Pure Error	3.75	4	0.9370			
**Cor Total**	1550.14	12				
**Std. Dev.**	5.89		**R^2**	0.8434		
**Mean**	81.88		**Adjusted R^2**	0.7315		
**C.V. %**	7.19		**Predicted R^2**	-0.5735		
			**Adeq Precision**	8.0517		

(5) Naringin

Source	Sum of Squares	df	Mean Square	F-value	p-value	
**Quadratic model**	1192.15	5	238.43	9.56	0.0049	significant
A-THF ratio	396.91	1	396.91	15.91	0.0053	
B-NaCl ratio	211.23	1	211.23	8.47	0.0227	
AB	153.76	1	153.76	6.16	0.0420	
A^2	13.54	1	13.54	0.5426	0.4853	
B^2	305.30	1	305.30	12.24	0.0100	
**Residual**	174.63	7	24.95			
Lack of Fit	170.42	3	56.81	54.00	0.0011	significant
Pure Error	4.21	4	1.05			
**Cor Total**	1366.78	12				
**Std. Dev.**	4.99		**R^2**	0.8722		
**Mean**	82.22		**Adjusted R^2**	0.7810		

**C.V. %**	6.08		**Predicted R^2**	-0.2740		
			**Adeq Precision**	8.4482		

(6) Hesperidin

Source	Sum of Squares	df	Mean Square	F-value	p-value	
**Quadratic model**	1241.16	5	248.23	8.99	0.0059	significant
A-THF ratio	416.67	1	416.67	15.09	0.0060	
B-NaCl ratio	130.67	1	130.67	4.73	0.0661	
AB	210.25	1	210.25	7.61	0.0281	
A^2	12.79	1	12.79	0.4630	0.5181	
B^2	349.66	1	349.66	12.66	0.0092	
**Residual**	193.34	7	27.62			
Lack of Fit	189.58	3	63.19	67.23	0.0007	significant
Pure Error	3.76	4	0.9400			
**Cor Total**	1434.50	12				
**Std. Dev.**	5.26		**R^2**	0.8652		
**Mean**	80.90		**Adjusted R^2**	0.7689		
**C.V. %**	6.50		**Predicted R^2**	-0.3465		
			**Adeq Precision**	8.7291		

(7) Diosmin

Source	Sum of Squares	df	Mean Square	F-value	p-value	
**Quadratic model**	1722.13	5	344.43	8.81	0.0063	significant
A-THF ratio	657.31	1	657.31	16.81	0.0046	
B-NaCl ratio	42.67	1	42.67	1.09	0.3310	
AB	342.25	1	342.25	8.75	0.0212	
A^2	63.36	1	63.36	1.62	0.2437	
B^2	397.03	1	397.03	10.15	0.0154	

**Residual**	273.74	7	39.11			
Lack of Fit	268.61	3	89.54	69.79	0.0007	significant
Pure Error	5.13	4	1.28			
**Cor Total**	1995.87	12				
**Std. Dev.**	6.25		**R^2**	0.8628		
**Mean**	80.14		**Adjusted R^2**	0.7649		
**C.V. %**	7.80		**Predicted R^2**	-0.3731		
			**Adeq Precision**	9.2991		

(8) β-Cryptoxanthin

Source	Sum of Squares	df	Mean Square	F-value	p-value	
**Quadratic model**	2205.37	5	441.07	19.38	0.0006	significant
A-THF ratio	1296.54	1	1296.54	56.96	0.0001	
B-NaCl ratio	406.73	1	406.73	17.87	0.0039	
AB	44.22	1	44.22	1.94	0.2060	
A^2	79.72	1	79.72	3.50	0.1035	
B^2	212.54	1	212.54	9.34	0.0184	
**Residual**	159.34	7	22.76			
Lack of Fit	108.63	3	36.21	2.86	0.1684	not significant
Pure Error	50.71	4	12.68			
**Cor Total**	2364.71	12				
**Std. Dev.**	4.77		**R^2**	0.9326		
**Mean**	73.89		**Adjusted R^2**	0.8845		
**C.V. %**	6.46		**Predicted R^2**	0.5093		
			**Adeq Precision**	14.1509		

(9) Campesterol

Source	Sum of Squares	df	Mean Square	F-value	p-value	

**Quadratic model**	3480.33	5	696.07	175.00	< 0.0001	significant
A-THF ratio	154.03	1	154.03	38.73	0.0004	
B-NaCl ratio	2293.21	1	2293.21	576.56	< 0.0001	
AB	234.09	1	234.09	58.85	0.0001	
A^2	73.69	1	73.69	18.53	0.0035	
B^2	467.88	1	467.88	117.63	< 0.0001	
**Residual**	27.84	7	3.98			
Lack of Fit	25.01	3	8.34	11.77	0.0187	significant
Pure Error	2.83	4	0.7080			
**Cor Total**	3508.18	12				
**Std. Dev.**	1.99		**R^2**	0.9921		
**Mean**	82.28		**Adjusted R^2**	0.9864		
**C.V. %**	2.42		**Predicted R^2**	0.9282		
			**Adeq Precision**	42.0566		

(10) Stigmasterol

**Source**	**Sum of Squares**	**df**	**Mean Square**	**F-value**	**p-value**	
**Quadratic model**	4397.73	5	879.55	39.73	< 0.0001	significant
A-THF ratio	249.61	1	249.61	11.27	0.0121	
B-NaCl ratio	2760.61	1	2760.61	124.69	< 0.0001	
AB	556.96	1	556.96	25.16	0.0015	
A^2	33.47	1	33.47	1.51	0.2586	
B^2	571.20	1	571.20	25.80	0.0014	
**Residual**	154.97	7	22.14			
Lack of Fit	144.72	3	48.24	18.82	0.0080	significant
Pure Error	10.25	4	2.56			
**Cor Total**	4552.71	12				
**Std. Dev.**	4.71		**R^2**	0.9660		

**Mean**	83.71		**Adjusted R^2**	0.9416		
**C.V. %**	5.62		**Predicted R^2**	0.6866		
			**Adeq Precision**	20.8035		

(11) Sitosterol

**Source**	**Sum of Squares**	**df**	**Mean Square**	**F-value**	**p-value**	
**Quadratic model**	2616.55	5	523.31	41.19	< 0.0001	significant
A-THF ratio	127.88	1	127.88	10.06	0.0157	
B-NaCl ratio	1686.73	1	1686.73	132.75	< 0.0001	
AB	418.20	1	418.20	32.91	0.0007	
A^2	13.51	1	13.51	1.06	0.3367	
B^2	268.62	1	268.62	21.14	0.0025	
**Residual**	88.94	7	12.71			
Lack of Fit	85.45	3	28.48	32.63	0.0029	significant
Pure Error	3.49	4	0.8730			
**Cor Total**	2705.49	12				
**Std. Dev.**	3.56		**R^2**	0.9671		
**Mean**	85.13		**Adjusted R^2**	0.9436		
**C.V. %**	4.19		**Predicted R^2**	0.6825		
			**Adeq Precision**	22.2921		

(12) β-Carotene

**Source**	**Sum of Squares**	**df**	**Mean Square**	**F-value**	**p-value**	
**Quadratic model**	4944.04	5	988.81	21.42	0.0004	significant
A-THF ratio	4144.88	1	4144.88	89.80	< 0.0001	
B-NaCl ratio	292.60	1	292.60	6.34	0.0399	
AB	0.0900	1	0.0900	0.0019	0.9660	
A^2	96.54	1	96.54	2.09	0.1914	

B^2	224.31	1	224.31	4.86	0.0633	
**Residual**	323.09	7	46.16			
Lack of Fit	87.11	3	29.04	0.4922	0.7066	not significant
Pure Error	235.98	4	58.99			
**Cor Total**	5267.14	12				
**Std. Dev.**	6.79		**R^2**	0.9387		
**Mean**	57.42		**Adjusted R^2**	0.8948		
**C.V. %**	11.83		**Predicted R^2**	0.7721		
			**Adeq Precision**	14.8872		
